# Teacher Characteristics and Perceptions of Pest Management Curricula: Clues to Adoption and Continuation

**DOI:** 10.3390/insects4020177

**Published:** 2013-04-15

**Authors:** Makena Mason, Maria Aihara-Sasaki, J. Kenneth Grace

**Affiliations:** College of Tropical Agriculture & Human Resources, University of Hawaii at Manoa, 3050 Maile Way, Gilmore Hall 310, Honolulu, HI 96822, USA; E-Mails: aiharasa@hawaii.edu (M.A.-S.); kennethg@hawaii.edu (J.K.G.)

**Keywords:** curriculum efficacy, curriculum adoption, integrated pest management, *Coptotermes formosanus*, termites, project evaluation, service-learning

## Abstract

*Educate to Eradicate* is a K-12 curriculum project using termite biology and control as the basis for science education that has been implemented in over 350 Hawaii public school classrooms. To encourage sustained implementation of the project, we aimed to identify factors that influence the adoption and continuation of pest management curricula in public school classrooms. Regression analysis of teacher survey data were used to create predictive models of teacher continuation. Teachers motivated by “*exciting students about science*”, who perceived increases in “*student understanding and comprehension of major termite knowledge concepts*” and/or students as “*more interested in termites after participating in this project*” were more likely to continue curriculum. Teachers who had worked at their current school over 21 years at the time of curriculum adoption, and/or who identified having subject specialties not listed on the survey were less likely to continue curriculum. Additionally, teachers servicing lower socioeconomic level students were less likely to continue the curricula.

## 1. Introduction

*Educate to Eradicate* is a K-12 curriculum project using termite biology and management as the basis for science education that has been implemented in over 350 Hawaii public school classrooms with more than 12,530 students and is coupled with community outreach efforts [1,2]. As part of the curricula, students assemble and observe live termite habitats. This activity dovetails with lessons covering data collection, predictions, and inquiry. Communication, interdependence, and adaption are explored. Subsequent lessons and investigations use a range of grade-appropriate pedagogies to further reinforce these concepts, while introducing termite lifecycles, prevention, and control [2].

The University of Hawaii Termite Project has recruited teachers by offering professional development credits, curriculum/materials, and in-class curriculum modeling. The project was advertised by flyer-drops and word of mouth. Teachers self-selected into the curriculum and underwent weekend, weeknight, and/or on-site professional development. Breadth of training varied based on teacher preference. Typically, teachers received both direct instruction on and in-class modeling of curriculum during the first year of partnership. In subsequent years, teachers implemented curriculum independently or co-taught with project staff. Despite continued growth in the number of teachers joining the program and the number of classrooms implementing the curriculum, over six years of the project (2004–2010), nearly two-thirds of teachers did not repeat the curriculum after the first year of implementation.

Past science curriculum studies have linked teacher age, gender, experience, and tenure [3] to novel science curriculum adoption rates. Teachers’ content knowledge and attitude were positively correlated with the implementation of state-sponsored curriculum [3]. Simplicity and trial-ability of curricula have been predictive of intended continuation. Additional factors associated with teacher adoption and continuation of curriculum include perceived resource availability, quality of professional development, faculty support, standard alignment, student abilities, planning time, and technical support [4,5,6,7,8,9].

This study aimed to identify factors that influenced the adoption and continuation of pest management curricula in public school classrooms. Prior to the study, project staff believed classroom experience, science training, and grade level would be important predictors of teacher continuation. In order to focus our teacher recruitment efforts and achieve sustained adoption of curricula, we examined past partner teacher survey responses for clues to teachers’ motives for project continuation. 

## 2. Materials and Methods

All *Educate to Eradicate* partner teachers were targeted for project evaluation throughout curricula implementation. Partner teacher demographics and perceptions of *Educate to Eradicate* curricula were recorded with surveys. To ensure content validity, survey tools were reviewed by a panel of experts from: (1) the University of Hawaii Termite Project, Department of Plant and Environmental Protection Sciences; (2) the Curriculum Research and Development Group, University of Hawaii at Manoa College of Education; and (3) Graduate Science, Technology, Engineering, and Mathematics (STEM) Fellows in K-12 Education Program, University of Hawaii at Manoa. Surveys were designed to capture teachers’ motivations, subject specialties, teaching experience, and education through multiple-choice and open-ended questions. Surveys also measured perceptions of curricula, resources, and impacts on teacher/student learning with Likert-type scales. Two surveys were created for elementary (version A) and middle/high school (version B) teachers. Initially, these surveys were created largely for project evaluation. Here, we further examine survey data for clues to teacher continuation.

Immediately after unit completion, all teachers were asked to return surveys (from 2004 to present). Teachers who omitted surveys were reminded within one month, through email. When teachers continued use of the curriculum and submitted several surveys over successive years, the first submission was used for analysis, as representative of their initial impressions. Omitted responses to survey questions were treated as missing values and not included in response means. Response frequencies and means were calculated.

Linear regression (stepwise method, *P* < 0.1 enter rate) was used to correlate teacher continuation (participated: 0- one year, 1-two years, 2- three or more years) on the basis of grade level, science background, teaching experience, perceptions of project content/pedagogy, motivations, subject specialties, and school socioeconomic status (SES) (SAS software, Version 9.2, linear regression). Teacher continuation data were square root transformed [√(X + 0.5)] because data points are small whole-number counts of rare events. School SES data were arcsine transformed because they were reported as a percent of the student population. Motivators and subject specialties were coded as quantitative (0 = not selected or 1 = selected). Survey data were collected with two surveys (versions A and B). Regression analysis was applied to demographic and teacher motivator questions for all responders. Perceptions of curriculum were analyzed separately for survey versions A and B, due to different constructs, wordings, and scales. A total of three stepwise regressions were preformed (combined A and B, survey A, survey B).

## 3. Results

**Figure 1:**
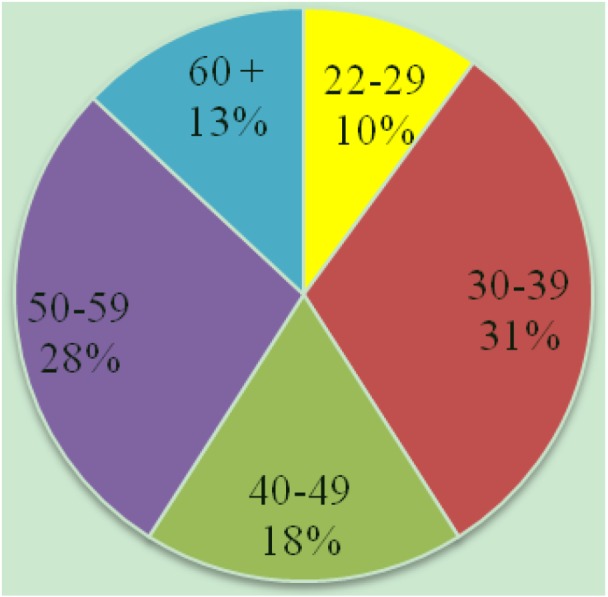
*Educate to Eradicate* partner teacher age distribution.

Participating teachers from 17 public schools on the islands of Oahu (12) and Maui (5) responded. Data collected from 2004–2010 were used for this analysis. Survey data from 66 (33%) partner teachers (88% female, 12% male) were collected. Forty-seven percent of the respondents continued the curriculum into at least a second year, 39% repeated curriculum three or more years, and 53% did not repeat curriculum. Higher continuation rates were seen within survey respondents, compared to the overall teacher population. Age distribution of the partner teachers is illustrated in Figure 1. The majority of *Educate to Eradicate* partner teachers had worked in Hawaii throughout their careers, and had moved between schools (Table 1).

**Table 1:** *Educate to Eradicate* partner teacher professional experience.

	≤5 years	6–10 years	11–20 years	21+ years
Years of teaching experience:	32%	16%	31%	21%
Years teaching in Hawaii:	34%	18%	30%	18%
Years at current school:	58%	15%	22%	5%

Teachers cited meeting science standards (88%), teaching science skills (76%), and using live insects (76%) most often as motivators for partnering with the University of Hawaii Termite Project (Table 2). Teachers who indicated that to “*excite students about science*” was a motivator were significantly more likely to continue curriculum (Table 3; *β* = 0.31, *P* = 0.007). Partner schools’ population of students who qualify for free or reduced lunch ranged from 9.4% to 89.2%. This measure of student socioeconomic status (SES) was a significant predictor of teacher continuation. Partner teachers servicing lower SES students are less likely to continue curriculum (*β* = −0.55, *P* = 0.045). Additionally, teachers who had worked at their current school over 21 years at the time of curriculum adoption were less likely to continue curriculum (*β* = −0.32, *P* = 0.039).

**Table 2:** *Educate to Eradicate* partner teacher motivations for adopting curriculum.

Motivations for partnering with the University of Hawaii Termite Project: *Educate to Eradicate*	Percent Response
Meet science standards	88%
Science skills	76%
Live insect observations	76%
Excite students about science	71%
Experience scientific inquiry	65%
Curriculum/ material resources	59%
Subject relevance to students’ lives	58%
Termite content knowledge	53%
University partnership	45%
In class termite staff support	38%
Community outreach	33%
Personal interest	33%
Language art/ reading skills	26%
Other responses:	9%
Tie in with community curriculum (6%). Contact with real scientists, Proximity to University- can walk to termite lab for field trip (1.5%). Part of grade level curriculum (1.5%).	

**Table 3:** Regression of 66 *Educate to Eradicate* partner teachers’ curriculum continuation on teacher characteristics (surveys A and B).

Variable	*β*	t-val	t-prob	Seq R^2^	Model R^2^
Motivation: *Excite students about science*	0.31	2.78	0.0074	0.12	0.12
Arcsine (percent free/reduced lunch)	−0.55	−2.05	0.045	0.067	0.18
21+ years at current school	−0.32	−2.12	0.039	0.058	0.24

Teachers reported that curriculum implementation was a valuable experience for teachers and students and had a significant impact on science learning, skills, and content knowledge (Table 4). The overall student/teacher learning experience was ranked between good to excellent (3.6). Mean teacher responses indicate good to excellent perceptions of project curriculum, resources, and impacts on teacher/student learning. The highest mean scores were for “*student enthusiasm and enjoyment levels during program participation*” (3.8) and “*overall impression of UH Termite Project curriculum, materials, and resources*” (3.7). Teachers’ response to the prompt “*student understanding and comprehension of major termite knowledge concepts*” was the only response positively correlated to teacher continuation (Table 5; *β* = 0.61, *P* < 0.0001). Teachers who identified having unlisted subject specialties were less likely to continue the curriculum (*β* = −0.48, *P* = 0.0003).

**Table 4:** *Educate to Eradicate* partner teachers’ perceptions of curriculum (survey A).

**Part 1: Project Curriculum and Resources**	**Mean**	**SD**
1. Overall impression of UH Termite Project curriculum, materials, and resources	3.7	0.45
2. Arrangement of learning sequence and termite content knowledge topics	3.6	0.49
3. Classroom delivery of subject content and activities	3.5	0.58
4. Grade level appropriateness of content, activities, and worksheets	3.6	0.70
5. Activity and lesson alignment with Hawaii State Science Standards	3.4	0.64
6. Ability of the Termite Project Unit to fit into and enhance already existing classroom science curriculum	3.4	0.64
7. Effectiveness of visual aides and activities in demonstrating and enforcing termite content knowledge	3.6	0.49
4 = Excellent, 3 = Good, 2 = Satisfactory, 1 = Unsatisfactory		
**Part 2: Impact on Teacher/Student Learning**	**Mean**	**SD**
1. Overall student/ teacher learning experience with the UH Termite Project	3.6	0.58
2. Student enthusiasm and enjoyment levels during program participation	3.8	0.43
3. Did students show interest in learning about termites?	Yes	0.00
4. Student understanding and comprehension of major termite knowledge concepts	3.4	0.50
5. Were students excited about sharing their new termite knowledge with others (sharing knowledge project)?	Yes	0.00
6. Impact of program on science learning, skills, and content knowledge for both teacher and students	Significant	0.00
7. Was this a valuable experience for both teachers and students?	Yes	0.00
8. Would you consider partnering with the project again next school year?	Yes	0.00
4 = Excellent, 3 = Good, 2 = Satisfactory, 1 = Unsatisfactory [Questions 3, 5–8 are binary response]		

**Table 5:** Regression of 43 *Educate to Eradicate* partner teachers’ project continuation on perceptions of curriculum and teacher characteristics (survey A).

Variable	*β*	t-val	t-prob	Seq R^2^	Model R^2^
Impact on teacher/ student learning: *Student understanding and comprehension of major termite knowledge concepts*	0.61	6.62	<0.0001	0.44	0.44
Teacher subject specialty: *Other*	−0.48	−4.34	0.0003	0.34	0.77

Teachers indicated that students gained knowledge (4.5, 4.4) and the ability to recognize termite infestation (3.8) through project participation (Table 6). Teachers *agree* or *strongly agree* that the project increased student interest and awareness of termites (4.6). Students were motivated during the project and teachers indicated they would be willing to conduct the project again (4.6). On average, teachers were *unsure* or *agreed* that the project encouraged community outreach from students (18% Strongly Agree, 32% Agree, 36% Unsure, 14% Disagree). Overall, teachers were more confident about the project’s ability to help connect parents with the learning program (13% Strongly Agree, 57% Agree, 21.5% Unsure, 8.5% Disagree). Teachers who perceived students as “*more interested in termites after participating in this project*” were more likely to continue curriculum (Table 7; *β* = 0.25, *P* = 0.014).

**Table 6:** *Educate to Eradicate* partner teacher perceptions of curriculum (survey B).

Impact on Student Learning	Mean	SD
1. My students gained knowledge about termites from this project	4.5	0.79
2. My students gained awareness of termites through this project	4.6	0.72
3. This project helped my students understand the nature of the scientific process	4.0	0.82
4. My students were motivated during the project	4.6	0.72
5. My students gained content knowledge through this project	4.4	0.72
6. This project helped us connect parents with the learning program	3.7	0.81
7. My students could recognize signs of termite infestation at our school	3.8	0.96
8. I would be willing to conduct this project in my classroom again	4.6	0.72
9. My students are more interested in termites after participating in this project	4.6	0.72
10. My students are more interested in science after participating in this project	4.2	0.90
11. This project encouraged community outreach from my students	3.5	0.96
12. I was able to easily incorporate this project into my curriculum	4.3	0.85
13. The lessons and learning sequence was appropriate for my students	4.3	0.71
14. The program was well-aligned to the Hawaii State Science Standards	4.3	0.62
15. The visuals and activities provided were effective in demonstrating and reinforcing termite content knowledge	4.5	0.79
5 = Strongly Agree, 4 = Agree, 3 = Unsure, 2 = Disagree, 1 = Strongly Disagree		

**Table 7:** Regression of 23 partner teachers’ project continuation on perceptions of curriculum and teacher characteristics (survey B).

Variable	*β*	t-val	t-prob	Seq R^2^	Model R^2^
Impact on student learning: *My students are more interested in termites after participating in this project*	0.25	2.70	0.014	0.26	0.26

## 4. Discussion

Teachers reported positive outcomes as a result of project implementation. Teachers positively rated curriculum effects on student/teacher learning, student motivation/interest/enthusiasm, and awareness. With these glowing reviews, why do so many teachers fail to continue the *Educate to Eradicate* curriculum? All teachers agreed they would be willing to implement the project the following year. What could account for the gap between teachers’ stated willingness to continue their partnership with the project and the reality that only one out of three teachers do so? It is possible that this contradiction reflects social desirability effects or the lack of anonymity in the survey. Regression analysis indicated the motivation to “*excite students about science*” was correlated with teacher continuation. Rates of novel computer science curriculum adoption have also been predicted by teachers’ desire to excite students (*β* = 0.70) [5]. Teachers with positive perceptions of “*impacts on student understanding and comprehension of major termite knowledge concepts*” and “*students are interested in termites after participating in this project*” were more likely to continue the curriculum. Positive attitudes toward curricula [3] and perceived student ability [6] have been linked to curricula adoption in past studies. 

A similarly structured study found that as a teacher’s tenure at a school increased, the likelihood of adopting the state-sponsored agriscience curriculum decreased (*β* = −0.25, *P* < 0.01) [3]. We found teachers with over 21 years at a school (*β* = −0.32, *P* = 0.039) were less likely to continue curricula. Perhaps teachers fail to adopt new curricula later in their careers, due to habitualized instruction or pending retirement. Teachers at schools with large percentages of students eligible for free or reduced school lunches were less likely to continue curriculum. This may be associated with higher rates of teacher attrition at schools with lower socioeconomic status [10] or an increased emphasis on standardized testing [11]. 

We suggest further examination of barriers to implementation including: alignment with instructional standards, department-wide buy-in [5], planning time, technology support, and quality of professional development [7]. A more robust methodology will be required to fully render the gestalt of teacher curricula adoption.

## 5. Conclusions

Teacher self-reports of intended curriculum use did not accurately predict continuation. Some survey data could be used to create modest predictive models. Teachers who used the curriculum to excite students about science and perceived an increase in students’ interest, knowledge, and comprehension of termite concepts were more likely to continue the project within their classrooms. Teachers who had lengthy tenure at one school, who worked at lower SES schools, or who had unique subject specialties were less likely to continue. A deeper investigation of barriers to curricula implementation is needed.
